# Visualizing Time-Varying Effect in Survival Analysis: 5 Complementary Plots to Kaplan-Meier Curve

**DOI:** 10.1155/2022/3934901

**Published:** 2022-03-29

**Authors:** Qiao Huang, Chong Tian

**Affiliations:** ^1^Tongji Medical College, Huazhong University of Science and Technology, Wuhan, China; ^2^Institute of Health Policy and Management (Think Tank), Huazhong University of Science and Technology, Wuhan, China; ^3^School of Nursing, Tongji Medical College, Huazhong University of Science and Technology, China

## Abstract

**Background:**

Kaplan-Meier (KM) curve has been widely used in the field of oxidative medicine and cellular longevity. However, time-varying effect might be presented in KM curve and cannot be intuitively observed. Complementary plots might promote clear insights in time-varying effect from KM curve.

**Methods:**

Three KM curves were identified from published randomized control trials: (a) curves diverged immediately; (b) intersected curves with statistical significance; and (c) intersected curves without statistical significance. We reconstructed individual patient data, and plotted 5 complementary plots (difference in survival probability and risk difference, difference in restricted mean survival time, landmark analyses, and hazard ratio over time), along with KM curve.

**Results:**

Entanglement and intersection of two KM curves would make the 5 complementary plots to fluctuate over time intuitively. Absolute effects were presented in the 3 plots of difference in survival probability, risk, and restricted mean survival time. Changed *P* values from landmark analyses were used to inspect conditional treatment effect; the turning points could be identified for further landmark analysis. When proportional hazard assumption was not met, estimated hazard ratio from traditional Cox regression was not appropriate, and time-varying hazard ratios could be presented instead of an average and single value.

**Conclusions:**

The 5 complementary plots with KM curve give a broad and straightforward picture of potential time-varying effect. They will provide clear insight in treatment effect and assist clinicians to make decision comprehensively.

## 1. Introduction

Multiple myeloma is a neoplastic disease of plasma cell characterized by the accumulation of clonal cells in the bone marrow. These plasma cells overproduce intracellular reactive oxygen species (ROS), resulting in unbalanced redox homeostasis [1]. Unbalanced production of ROS leads to oxidative stress, and the oxidative stress signaling could contribute to acquired melphalan resistance [2]. Antioxidant defense endowed multiple myeloma cells with resistance to high-dose melphalan [3]. Many therapies, including bortezomib and melphalan, have been studied in many trials for untreated multiple myeloma [4, 5]. In these trials, time-to-event (TTE) data was collected, including survival time (until the occurrence of an event of interest, for example, death and progression of multiple myeloma) and status at last observation [6]. KM curve becomes an essential part in generating evidence-based information on TTE data and has been used for more than 70 years [7]. Previous studies have presented the application of Kaplan-Meier (KM) curve to analyze TTE data from oxidative medicine [8]. Time-specific survival probability can be estimated from KM curves and median survival time when survival probability drops to 50% or below [9, 10].

KM curve with two groups can be presented with various forms. For example, two curves separate widely from the start to the end of follow-up, or they can track closely at the early stage and separate at the end, or they can crossover at the early stage or at late stage. Our brains have been trained to chronically focus on the rate of decline between groups; however, many useful information may be ignored [11]. The diversity of KM curve makes it difficult to understand TTE data directly and comprehensively, especially when two survival curves intersect. To have a better understanding of KM curve directly, a transformation of KM curve into difference in survival probability and risk difference can been introduced as absolute measures, which have been highly recommended for clinical decision in comparative studies because of their intuition and practical value [12].

Log-rank test has been widely used to compare two survival curves between two groups. In addition, landmark analysis has been proposed to assess conditional association between treatment and survival outcome before and after the landmark [13]. A sensitivity analysis on different landmark times has been recommended. The magnitude of treatment effect, hazard ratio (HR), should be estimated using Cox proportional hazard regression [14, 15]. A key assumption in Cox regression is that the ratio of hazard functions does not vary with time, which is known as proportional hazard (PH) assumption. Interpretation of a single value of HR from Cox regression may be a challenge when PH assumption is not satisfied. Restricted mean survival time (RMST) is estimated by calculating the area under the survival curve between 0 and prespecific time (*τ*), and difference in RMST between two group has been promoted as an alternative statistic than HR without limitation of PH assumption [16–18].

In this study, we aimed to combine the four measures, including difference in survival probability over time, risk difference over time, difference in RMST over time, HR over time, and landmark analyses based on a series of time points, to provide a comprehensive information on treatment effect, which might help clinicians and lay people to understand treatment effect in TTE data more clearly.

## 2. Methods

### 2.1. Data Source

We identified an appropriate study based on the following inclusion criteria: (a) trials related to bortezomib, melphalan, and prednisone in multiple myeloma; (b) trials enrolling only two groups; (c) study reporting number of individuals at risks under KM curve; and finally, two appropriate studies with 3 different types of KM curves were included as our examples [4, 5].

**Figure 1:**
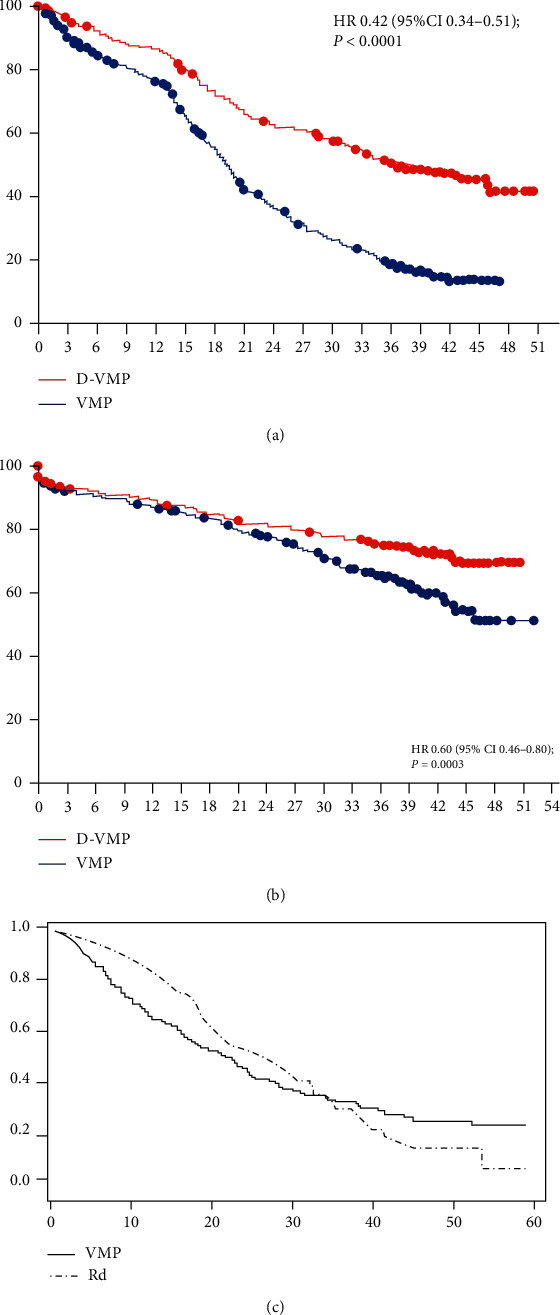
Three types of Kaplan-Meier curves.

In Mateos et al.'s study, the effect of daratumumab in combination with bortezomib, melphalan, and prednisone (D-VMP, *N* = 350) was compared with bortezomib-melphalan-prednisone (VMP, *N* = 356, control group). In the KM curve of progression-free survival (PFS), two curves diverged immediately and showed significant difference (Figure 1(a)). For the overall survival (OS), two survival curves intersected in the early stage and then diverged widely (Figure 1(b)). In Gentile et al.'s study, VMP (*N* = 257) and lenalidomide and low-dose dexamethasone (Rd, *N* = 222, control group) were extracted from two randomized phase III trials [19–21]. Treatment effect on PFS was evaluated and visualized; the two survival curves are intersected at a point in the medium term (Figure 1(c)).

### 2.2. Reconstruction of Individual Patient Data

A *R* function has been developed to reconstruct individual patient data (IPD) from published KM curve [22]. KM curves and total numbers of events in two groups were captured from included trials, and Engauge Digitizer was used to extract data points from KM curves [23].

### 2.3. Statistical Analysis

We replotted the KM curve based on corresponding IPD. Intervention group was marked in red, and control group was marked in gray. Proportional hazard assumption was tested using scaled Schoenfeld residuals; HR and corresponding 95% confidence interval (CI) were calculated based on Cox regression used in the original study; these data were annotated on the replotted KM curve. We also compared the reconstructed KM curve and estimated HR with those from original study to verify accuracy of reconstructed IPD.

Three absolute effects were visualized. Firstly, change of difference in survival probability over follow-up time was drawn. Survival probability and its standard error (SE) at a series of time points on both curves (intervention group and control group) were estimated using nonparametric Kaplan-Meier method. Difference in survival probability could be calculated, and 95% confidence interval of curve was constructed using Altman's method [24]. Secondly, change of risk difference over follow-up time was plotted. The numbers at risk decreased during follow-up; risk difference cannot be estimated based on 2×2 contingency table. Pseudovalue method was proposed to estimate risk difference in survival analysis, which was not limited to PH assumption [25]. Generalized estimating equation (GEE) was applied in pseudovalue regression to model the effects of covariates on risk of event. To explore arbitrary nonlinear relationship between risk difference and follow-up time, a restricted cubic spline of time and its interaction with group was included in GEE. Lastly, change of difference in RMST and 95% confidence interval over follow-up time were plotted based on Kaplan-Meier curve.

We performed a series of landmark analyses based on different cutoffs of follow-up time. Based on the cutoff, the IPD data was divided into prelandmark and postlandmark part. Log-rank test was applied in both parts, and paired *P* values were presented with transformation (Y = −log_10_ (*P*)). Using the - log_10_ (0.05) as reference, any point above the reference line indicated statistical significance. For feasibility of analysis, the paired landmark analyses were not conducted when any group had less than 5 participants or no event. Meanwhile, the change in HR over time and 95% confidence interval were estimated based on restricted cubic spline.

Reconstruction of IPD, data analysis, and visualization were conducted by using R version 3.5.1 software (the R Foundation for Statistical Computing, Vienna, Austria) with dplyr, survival, survRM2, rms, ggplot2, and cowplot package. All R codes were accessible in the supplementary material. Statistical significance tests were conducted using a 2-sided type I error rate of 5%.

## 3. Results

**Figure 2:**
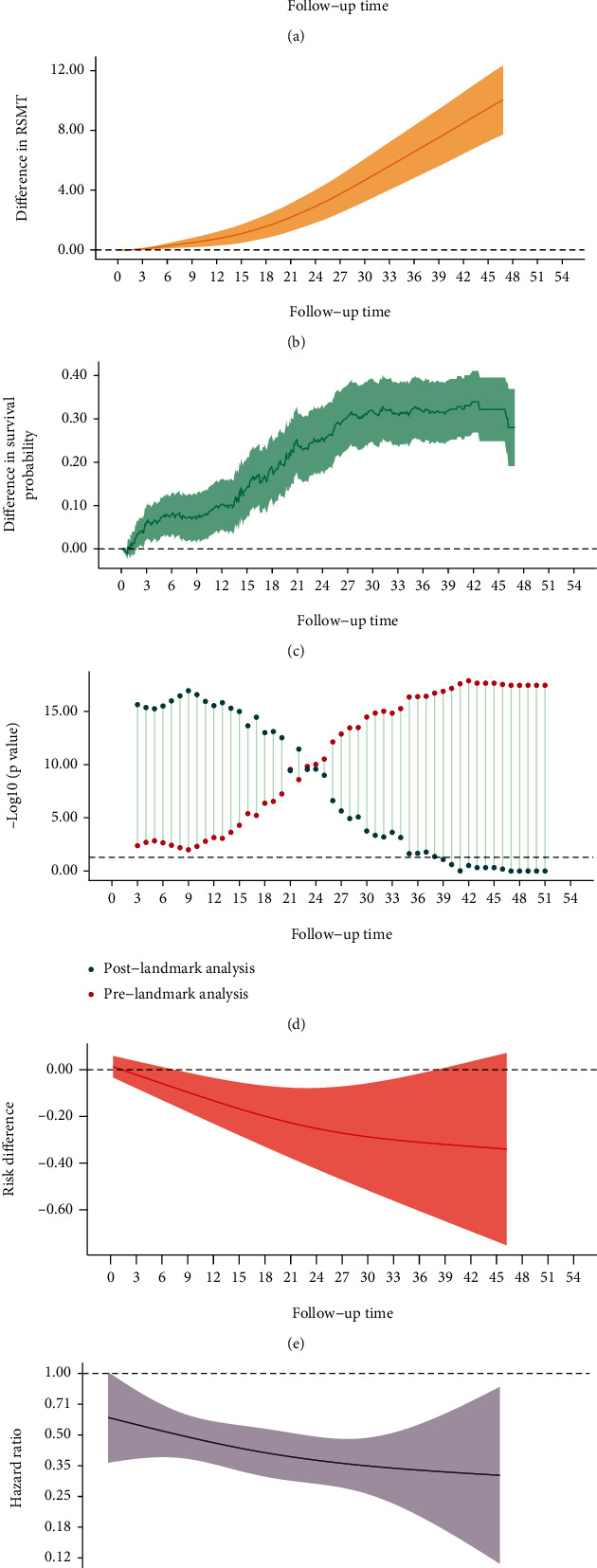
Two survival curves with significantly early and widening difference.

Generally, we expected that two survival curves diverged immediately and showed significant difference. In Figure 2 (PFS), the re-estimated HR is 0.42 (95% CI, 0.35, 0.52), which was similar to 0.42 (95% CI, 0.34 to 0.51), which supported high accuracy of reconstructed IPD (Figure 1(a) and Figure 2(a)). Intervention group always had a higher survival probability than control group during the whole follow-up period. The difference in survival probability (Figure 2(c)) between two groups increased slowly and entered into a plateau during the first 14 months. Subsequently, the difference increased sharply and then maintained stable. Plot of risk difference (Figure 2(e)) showed that the risk of event (progression) had been kept at a lower level in intervention group. Difference in RMST (Figure 2(b)) presented a steady upward trend, indicating that the intervention group always delayed the progression during the whole follow-up period because the 95% confidence band did not include 0. At 47 months, the difference in RMST between two group was about 10 months as the net profit. A “X” shape was presented in landmark analysis (Figure 2(d)); the *P* values from prelandmark parts were always significant (*P* < 0.05) and got smaller during follow-up, which indicated no apparent conditional association and supported the robustness of conclusion. We had observed a different divergence pattern before and after 14 months. Even though the PH assumption was hold, the corresponding *P* value was close to 0.05 and the HR decreased over time slightly, which suggested that the treatment effect increased over time (Figure 2(f)). Superiority of the intervention group to control group existed in both short and long term.

**Figure 3:**
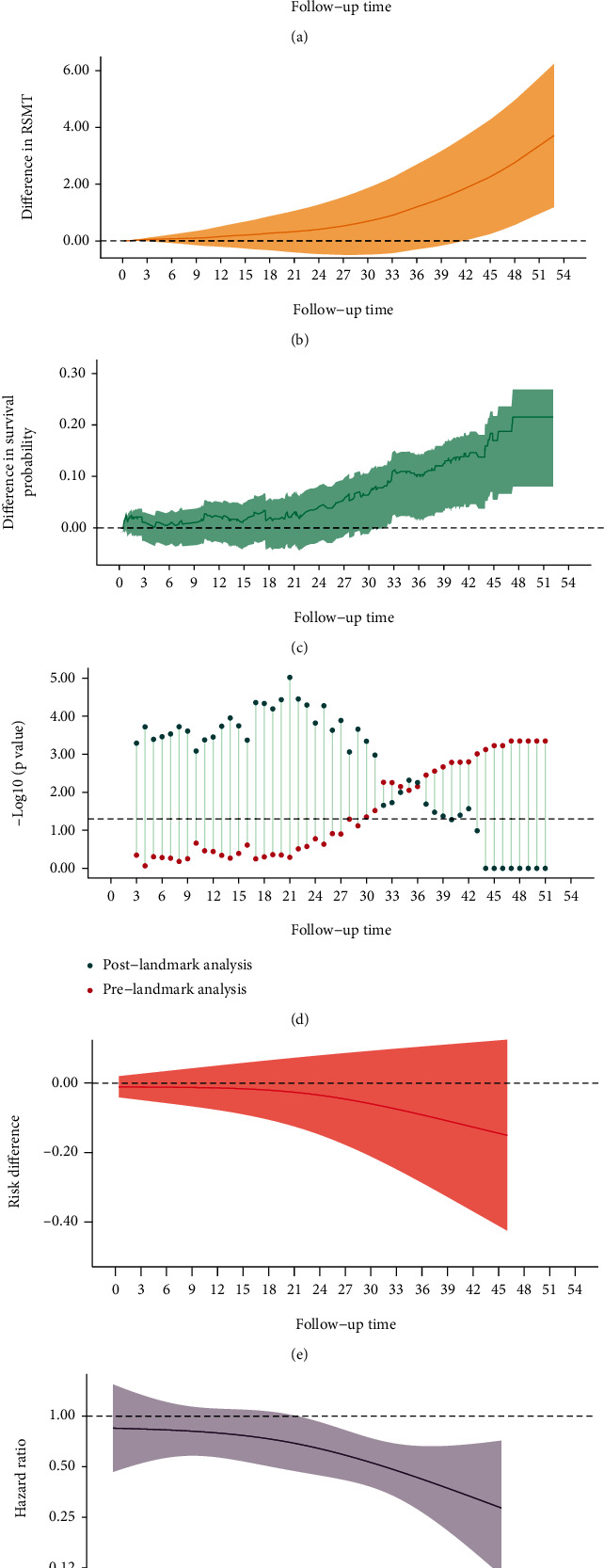
Two survival curves with intersection in the early stage and significant difference.

In Figure 2 (OS), two survival curves intersected in the early stage and then diverged widely (Figure 3(a)). The average HR was 0.61 (95% CI, 0.46, 0.81) which was similar to 0.60 (95% CI 0.46, 0.80) in raw plot (Figure 1(b)). The *P* value from PH assumption test was 0.041, suggesting time-varying treatment effect. Difference in survival probability (Figure 3(c)) kept stable in the first 21 months and then increased and peaked at 20% at 51 months. The risk difference plot (Figure 3(e)) presented similar change, and the turning point was also identified at 21 months. Both suggested that intervention had took effect after 21 months. A “J-shape” was found in the difference in RMST which increased slowly at first and then more rapidly (Figure 3(b)). After 40th month, the difference becomes statistically significant. Finally, patients in intervention group would significantly live about 3.8 months longer than those in control group during the 51 months. A X-shape was presented in the landmark analysis (Figure 3(d)). Before 28th month, all postlandmark analyses were significant, and all prelandmark analyses were not significant. The 21th month presented the smallest *P* value from postlandmark analysis. An apparent change in HR was found, which was consistent with the non-PH (Figure 3(f)). During the early stage, the treatment effect was not significant hazard. After 21 months, the HR was getting away from 1. The 21th month could be considered as a landmark time for further analysis.

**Figure 4:**
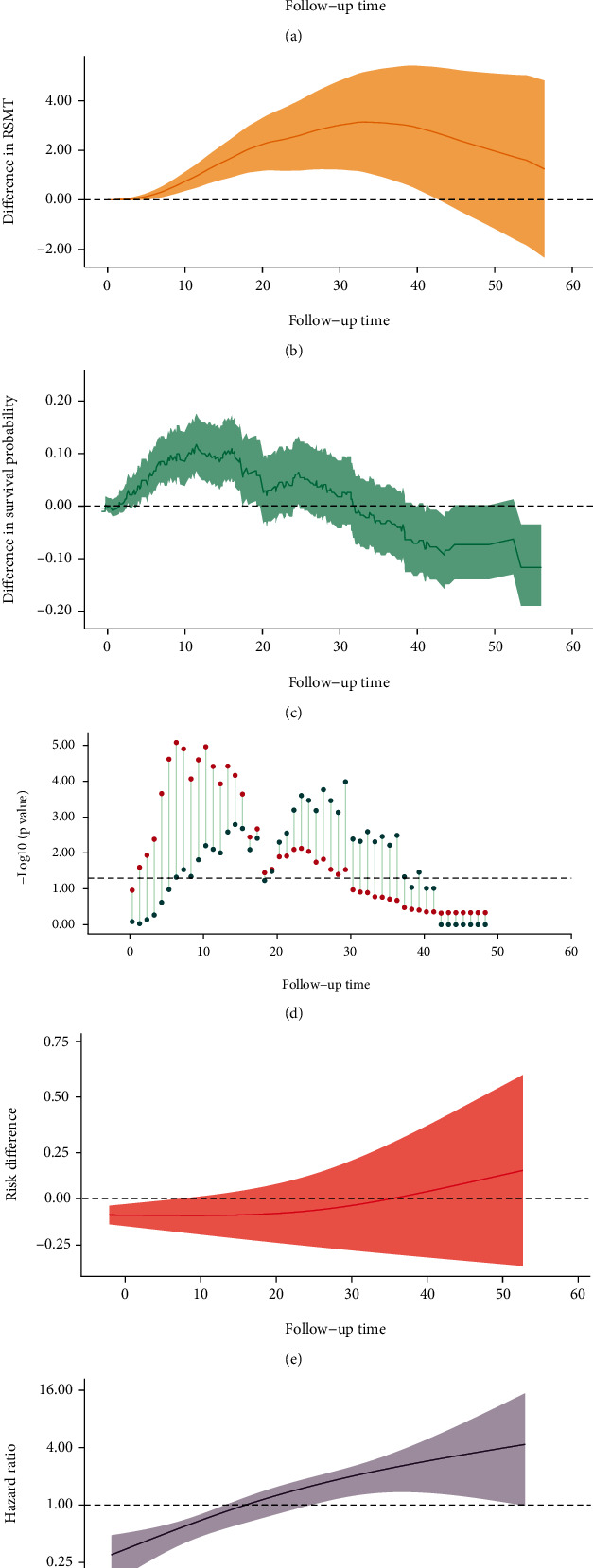
Two survival curves with divergence in the early stage and intersection in the later stage.

In Figure 4, two survival curves diverge in the early stage and intersect at the later stage (Figure 4(a)). The average HR was 0.94 with no statistical significance. The *P* value from PH assumption test was less than 0.001, suggesting time-varying effect. A reverse “U-shaped” change was found in the difference in survival probability (Figure 4(c)). At about 12 months, the difference reached the highest point (18%) with statistical significance. Subsequently, it decreased gradually and leveled out towards a minimum at -18%. The risk difference increased from negative value to positive value, suggested early benefit and late harm (Figure 4(e)). Another reverse “U-shaped” change was found in difference in RMST (Figure 4(b)). Treatment benefit in the early stage was diminished after about 32th month. Even though the benefit from treatment was not offset completely, the difference in RMST becomes insignificant at the end of 55 months. Both prelandmark and postlandmark analyses showed reverse “U-shaped” change which indicated a complex and entangling pre- and postrelationship (D). Between 12th month and 32th month, both prelandmark and postlandmark analyses reported statistical significances; a clinical and statistical landmark time could be determined. Non-PH could be validated in the changing HR over time (Figure B.). A tipping point at about 18th month instead of 32th month was observed; the intervention presented significant benefit on patients during early stage (HR<1); after that, the intervention effect appeared significant harm during late stage (HR<1). Based on B and F, the intervention did not benefit the patients comprehensively.

## 4. Discussion

Even though KM curves only presented falling trend, different falling forms generated various changes over time in survival probability, risk difference, difference in RMST, *P* values from prelandmark and postlandmark analyses, and HR. Magnitude of difference in survival probability and risk difference was estimated as projection of KM curve. RMST gave a cumulative treatment effect without limitation of PH assumption. Landmark analyses were used to identify the best landmark point where there was a clinical and significant difference between prelandmark and postlandmark part. Existence of entanglement and crossover caused these curves to fluctuate, and the significant turning points could be determined for further analysis, clinical explanation, and decision-making.

Frequently, *P* value from log-rank test or HR (95% confidence interval) was annotated on KM curve to provide more information for readers to make conclusions. HR was estimated from Cox proportional hazard regression as a relative measure of survival difference. Both practicing clinicians and lay people tended to overestimate effect size when only reporting relative measures [26, 27]. For example, the rate of event was 2‰ in intervention group and 1‰ in control group, which indicated an increase in risk by 100%. Absolute measures gave a better and intuitive presentation of actual situation, such as risk difference was only 1‰ in this case. However, absolute measures are influenced by baseline level which might vary across difference populations, resulting in less generalization than relative measures. Confusion about the efficacy and risk of treatment can be introduced in patients and clinicians when only reported one of them [28]. Two popular reporting specifications, consolidated standards of reporting trials (CONSORT) and strengthening the reporting of observational studies in epidemiology (STROBE), strongly urge researchers to report both measures whenever possible [29, 30]. Presentation of both relative and absolute effect size can provide a broad picture of the characteristics of treatment effect. However, a recent structural review showed that 75% reported only relative measures and 18% reported only absolute measures in the full texts of 344 published medical and public health articles [31]. In our study, we had estimated three kinds of absolute measures without limitation of PH assumption.

Change of difference in both survival probability and risk with time was to project the difference of two KM curves on the *x*-axis, which was convenient for observing and understanding the change intuitively. The number needed to treat (NNT) can be calculated by the reciprocal of absolute risk difference and indicates the number of treated subjects required to prevent one additional event [32]. Cumulative survival time presents special clinical significance for oncological studies. RMST as effect measure had attracted many attentions in survival analysis [17]. Difference in RMST indicates that patient will live longer (positive) or shorter (negative) and its magnitude presents the size of average gain or lost in life expectancy within prespecific time (*τ*).

Application of landmark analysis was a feasible method to explore possible conditional treatment effect [33]. Choice of landmark time is a crucial issue. Previous studies selected only one landmark time based on KM curve without additional explanation [34, 35]. However, a sensitivity analysis that performs landmark analysis using a series of time points was recommended to select appropriate time point [36]. In our study, we used the sensitivity analysis and combined the KM curve with landmark analysis with the same *x*-axis (follow-up time). Once a suitable landmark time was selected, a new KM curve partitioned by or after landmark time point can be presented [34, 37]. Meanwhile, the time point should be explained clinically and meaningfully. The change of valid sample size might influence the power of this analysis. All subjects were included in the prelandmark part; when the landmark time is specified in an earlier stage of follow-up, status of many subjects was marked as censoring, which resulted in a reduction in valid number of events. For postlandmark part, subjects whose events of interest occurred prior to the landmark time were excluded, sample size and statistical power for postlandmark analysis decreased.

Time-dependent covariate and time-dependent coefficient were two extensive applications of COX regression in clinical practice. When the exposure status was fixed but PH assumption was not hold, it suggested that the effect of exposure was not constant over the follow-up time, and the single HR value with average property was no longer appropriate. Time-varying HR was constructed using a special function of time HR (*t*) = *e*^*β*×*g*(*t*)^ [38]. Instantaneous effect of exposure can be obtained for any time point. In our study, we modified the implicit plot.cox.zph function in survival package and integrated it as a complementary plot. The turning point from HR<1 to HR>1 or from HR>1 to HR<1 could be found for clinical decision-making.

In the current study, we only applied comparison between two groups and developed *R* functions to visualize corresponding effect measures. When more than two treatment groups were included, KM plot and log-rank test were still feasible to evaluate overall effect; meanwhile, multiple comparisons were required for post-hoc analysis [39]. However, landmark analysis should be used with caution because points of prelandmark and postlandmark analysis might be presented redundantly. The differences in survival probability, risk, and RMST and change of HR over time should be separately estimated based on pairwise comparison; thus, we cannot obtain the overall effect. The developed *R* functions can be extended to accommodate multiple groups and visualize multiple lines from multiple comparisons. Moreover, falsely significant difference between any two groups should be addressed appropriately, and confidence interval should be adjusted accordingly.

This study had several strengths. 3 KM curves were selected as examples, and 5 complementary plots were illustrated to give a comprehensive perspective of time-varying effect. The KM curve and the 5 complementary plots were plotted with KM curves using a consistent *x*-axis for intuitive comparison. These were a number of limitations in this study: the exposure status was hypothesized to be fixed, and no time-dependent covariate was included. In addition, survival curves adjusted for covariates were not estimated; only time, status, and group were parameterized in developed *R* functions. Parametric survival regression and Cox proportional hazards model can be useful to estimate covariate-adjusted survival curves.

## 5. Conclusion

In this study, difference in survival probability over time, risk difference over time, difference in RMST over time, and landmark analyses using different cutoffs and HR over time were plotted with KM curves to give a complete and straightforward picture of potential time-varying effect in TTE data. They provide useful and straightforward adjuncts to, not a replacement for, KM curves and might help clinicians and lay people to understand treatment effect clearly and make decision smartly. Since integrated *R* functions had been developed, we hope that these 5 plots can also be reported as appurtenances to the KM curve in the future visualization of survival analysis.

## Data Availability

R code was provided as supplementary.

## Abbreviations

CONSORT: Consolidated standards of reporting trials
GEE: Generalized estimating equation
HR: Hazard ratio
IPD: Individual patient data
KM: Kaplan-Meier
NNT: Number needed to treat
PH: Proportional hazard
RMST: Restricted mean survival time
STROBE: Strengthening the reporting of observational studies in epidemiology
TTE: Time-to-event.

## Supplementary Materials

Supplementary Materials R functions to plot Kaplan-Meier curve and 5 complementary plots.
