# Supplementary material for: Visualizing Time-Varying Effect in Survival Analysis: 5 Complementary Plots to Kaplan-Meier Curve
Source: Oxid Med Cell Longev. 2022 Mar 29;2022:3934901. doi: 10.1155/2022/3934901 (PMC8983224; doi:10.1155/2022/3934901)
Supplement: Supplementary Materials — R functions to plot Kaplan-Meier curve and 5 complementary plots. [file 3934901.f1.pdf]

R functions

> **Visualizing time-varying treatment effect in survival analysis: 5 complementary plots to Kaplan-Meier curve**
>
> Version 1.0

R functions

- Function 1 : plotting Kaplan-Meier plot
- Function 2 : plotting difference in survival probability
- Function 3 : plotting risk difference using Psuedo-value regression
- Function 4: plotting difference in restricted mean survival time
- Function 5: plotting landmark analysis
- Function 6: plotting time-varying HR
- An example: Run 6 functions and combine the 6 plots

Function 1 : plotting Kaplan-Meier plot

```
KM_plot <- function(status,
                    time,
                    group = NULL,
                    data,
                    HR = TRUE,
                    HR.loc=c((as.integer(max(sfit$time)/6)),0.2),
                    PH=TRUE,
                    PH.loc=c((as.integer(max(sfit$time)/6)),0.4),
                    conf.level=0.95
                    ){
  #construct surv obj
  event <- deparse(substitute(status))
  time <- deparse(substitute(time))


  group <- deparse(substitute(group))
  fml <- paste0('Surv(', time, ",", event, ")~", group)
  sfit <- survfit(as.formula(fml), data)

  timeby = signif(max(sfit$time) / 6, 1)
  times <- seq(0, max(sfit$time), by = timeby)

  if (length(levels(summary(sfit)$strata)) == 0) {
    #no group information
    subs1 <- 1
    #store the number of timepoint stored in the timetable for survival curve
    subs2 <- 1:length(summary(sfit, censored = T)$time)
    #store the number of timepoint presented in number at risk table
    subs3 <-
      1:length(summary(sfit, times = times, extend = TRUE)$time)
  } else {
    subs1 <- 1:length(levels(summary(sfit)$strata))
```

```
  subs2 <- 1:length(summary(sfit, censored = T)$strata)
  subs3 <-
    1:length(summary(sfit, times = times, extend = TRUE)$strata)
}
Factor <- factor(summary(sfit, censored = T)$strata[subs2])

##################################
# data manipulation pre-plotting #
##################################
#Data to be used in the survival plot
df <- data.frame(
  time = sfit$time[subs2],
  n.risk = sfit$n.risk[subs2],
  n.event = sfit$n.event[subs2],
  n.censor = sfit$n.censor[subs2],
  surv = sfit$surv[subs2],
  strata = Factor,
  upper = sfit$upper[subs2],
  lower = sfit$lower[subs2]
)

###################################
# specifying axis parameteres etc #
###################################

p <-
  ggplot(df, aes(x = time,y = surv)) +
  geom_step(aes(color=strata),size = 0.75) +
  theme_bw() +
  theme(
    text = element_text(family = 'serif'),
    axis.title.x = element_text(family = 'serif', vjust = 0.7),
    panel.grid.minor = element_blank(),
    panel.grid.major = element_blank(),
    axis.line = element_line(size = 0.5, colour = "black"),
    legend.position = "none",
    legend.background = element_rect(fill = NULL),
    legend.key = element_rect(colour = NA),
    panel.border = element_blank(),
    axis.line.x = element_line(
      size = 0.5,
      linetype = "solid",
      colour = "black"
    ),
    axis.line.y = element_line(
      size = 0.5,
      linetype = "solid",
      colour = "black"
    )
  ) +
  scale_color_manual(values=c('#999999','#E61419'))+
  xlab(label='Follow-up time')+
  scale_y_continuous('Survival probability',
                     labels=function(x){sprintf("%.2f",x)})

#add HR and 95% CI
##construct function for extract HR and 95%CI to be presented in plot
extra.cox <- function(cox) {
```

```
    HR.CI <- paste0(
      "HR: ",
      round(cox$conf.int[1], 2),
      "(",
      round(cox$conf.int[3], 2),
      ", ",
      round(cox$conf.int[4], 2),
      ")"
    )
    cox.pvalue <- ifelse(cox$wald["pvalue"] < 0.001, "p<0.001",
                         paste('p=', round(cox$wald["pvalue"], 3)))
    return(paste0(HR.CI, ", ", cox.pvalue))
  }
  if (HR) {
    cox <- summary(coxph(as.formula(fml), data),conf.int=conf.level)
    HR.label <- extra.cox(cox)
    p <-
      p + annotate(
        'text',
        x = HR.loc[1],
        y = HR.loc[2],
        label = HR.label,
        family = 'serif',
        hjust = 0
      )
  }
  if (PH){
    ph.test <- cox.zph(coxph(as.formula(fml), data))$table
    ph.chisq <- round(ph.test[2], 3)
    ph.pvalue <- ifelse(ph.test[3]< 0.001, "p<0.001",
                         paste('p=', round(ph.test[3], 3)))
    PH.label <- paste0('Test for proportional hazard:\n Chis-quare:',
                       ph.chisq,', ',ph.pvalue)
    p <-
      p + annotate(
        'text',
        x = PH.loc[1],
        y = PH.loc[2],
        label = PH.label,
        family = 'serif',
        hjust = 0
      )
  }
  return(p)
}
```

Function 2 : plotting difference in survival probability

```
SurvDiff.surv <-
  function(time, status, group, data, interval = 10,converse=FALSE) {
    #construct surv obj
    event.char <- deparse(substitute(status))
```

```r
  time.char <- deparse(substitute(time))
  group.char <- deparse(substitute(group))
  fml <-
    as.formula(paste0('Surv(', time.char, ",", event.char, ")~", group.char))
  fit <- npsurv(fml, data = IPD)
  #constuct data of plot
  max <- fit$maxtime
  min <- min(0, fit$time)
  times <- sort(unique(c(fit$time, seq(min, max, by = interval))))

  f <- summary(fit, times = times)
  group.label <- levels(f$strata)

  point.se <- function(grp.label) {
    tf <- f$strata == grp.label
    tim <- f$time[tf]
    surv <- f$surv[tf]
    se <- f$std.err[tf]
    j <- match(times, tim)
    return(list(
      time = times,
      surv = surv[j],
      se = se[j]
    ))
  }

  if (converse) {
    a <- point.se(group.label[1])
    b <- point.se(group.label[2])
    print(paste0(group.label[1], '-', group.label[2]))

  } else{
    a <- point.se(group.label[2])
    b <- point.se(group.label[1])
    print(paste0(group.label[2], '-', group.label[1]))
  }
  #calculate survival difference and CI
  #Altman's methods based on S.E. of survival probability
  surv <- a$surv - b$surv
  se <- sqrt(a$se ^ 2 + b$se ^ 2)
  z <- qnorm((1 + 0.95) / 2)
  lo <- surv - z * se
  hi <- surv + z * se

  df <- data.frame(
    time = times,
    SD = surv,Lower = lo,Upper = hi
  )
  rtn <<- df[complete.cases(df),]
  ggplot(data = rtn, aes(x = time, y = SD)) +
    geom_line(size = .5, alpha = 1,color='#06A77D') +
    geom_ribbon(aes(x = time,
                    ymax = Upper,
                    ymin = Lower),
                alpha = 0.5,fill='#06A77D') +
    geom_hline(yintercept = 0,linetype='dashed')+
    xlab(label='Follow-up time')+
    scale_y_continuous('Difference in survival probability',
```

```
                          labels=function(x){sprintf("%.2f",x)})+
       theme_bw() +
        theme(
          text = element_text(family = 'serif'),
          axis.title.x = element_text(family = 'serif', vjust = 1.7),
          panel.grid.minor = element_blank(),
          panel.grid.major = element_blank(),
          axis.line = element_line(size = 0.5, colour = "black"),
          legend.background = element_rect(fill = NULL),
          legend.key = element_rect(colour = NA),
          panel.border = element_blank(),
          axis.line.x = element_line(
            size = 0.5,
            linetype = "solid",
            colour = "black"
          ),
          axis.line.y = element_line(
            size = 0.5,
            linetype = "solid",
            colour = "black"
          )
        )
}
```

Function 3 : plotting risk difference using Psuedo-value regression

```
RiskDiff.surv <- function(time,
                          status,
                          group,
                          data
                          )
 {
  #construct surv obj
  event.char <- deparse(substitute(status))
  time.char <- deparse(substitute(time))
  #group.char and fml must be a local variable when paralle
  group.char <- deparse(substitute(group))
  fml <-
    as.formula(paste0('Surv(', time.char, ",", event.char, ")~", group.char))
  data <- data

  # Pseudo-value method -----------------------------------------------------
    #create pseudo-value for next GEE estimation
    require(pseudo)
    print('---start to pseudo value---')
    time.tmp <- pseudosurv(time = data[, time.char],
                              event = data[, event.char])$time
    pseudo<- pseudosurv(time = data[, time.char],
                          event = data[, event.char],
                        tmax=seq(min(time.tmp),max(time.tmp),length.out=200))
    timelist <- pseudo$time
    #reshape data
    dat.gee <- NULL
    for (i in 1:length(timelist)) {
```

```
      dat.gee <- rbind(dat.gee,
                       cbind(
                         data,
                         pseudo = pseudo$pseudo[, i],
                         tpseudo = timelist[i],
                         id = 1:nrow(data)
                       ))
    }
    dat.gee <- dat.gee[order(dat.gee$id),]

    #GEE method
    gee.fit <- geese(
      # a restricted cubic splien to present non-linear relationship
      # only two variable included: time and group
      pseudo ~ rcs(tpseudo, 3) * as.factor(group),
      data = dat.gee,
      id = id,
      family = gaussian,
      mean.link = 'identity',
      jack=TRUE,
      corstr='independence'
    )
    splineforpre <- rcspline.eval(timelist,
                                  knots = attr(rcs(dat.gee$tpseudo, 3),
'parms'),
                                  inclx = TRUE)
    gee.sum <- summary(gee.fit)$mean[,c(1:2)]
    gee.ci <- t(apply(gee.sum,1,FUN=function(x)
{return(x[1]+1.96*c(-1,1)*x[2])}))

    #point estimation and 95% confidence interval
    rd.point <- -cbind(1, splineforpre) %*% gee.fit$beta[c(4, 5, 6)]
    rd.lower <- -cbind(1, splineforpre) %*% gee.ci[c(4,5,6),1]
    rd.upper <- -cbind(1, splineforpre) %*% gee.ci[c(4,5,6),2]

    rtn <- data.frame(time = timelist, RD = rd.point,
Lower=rd.lower,Upper=rd.upper)

  ##plot---------------------------------------------------------
  p <- ggplot(data = rtn, aes(x = time, y = RD)) +
    geom_line(size = 0.75,
              alpha = 1,
              color = '#EC0B43') +
    geom_hline(yintercept = 0, linetype = 'dashed') +
    xlab(label='Follow-up time')+
    scale_y_continuous('Risk Difference',
                       labels=function(x){sprintf("%.2f",x)})+
    theme_bw() +
    theme(
      text = element_text(family = 'serif'),
      axis.title.x = element_text(family = 'serif', vjust = 1.7),
      panel.grid.minor = element_blank(),
      panel.grid.major = element_blank(),
      axis.line = element_line(size = 0.5, colour = "black"),
      legend.background = element_rect(fill = NULL),
      legend.key = element_rect(colour = NA),
      panel.border = element_blank(),
      axis.line.x = element_line(
```

```
        size = 0.5,
        linetype = "solid",
        colour = "black"
      ),
      axis.line.y = element_line(
        size = 0.5,
        linetype = "solid",
        colour = "black"
      )
    )
  if(TRUE){
    p <- p+
      geom_ribbon(aes(
        x = time,
        ymax = Upper,
        ymin = Lower
      ),
      alpha = 0.5,fill='#EC0B43')    ##EC0B43
    return(p)
  }else{
    return(p)
  }
}
```

Function 4: plotting difference in restricted mean survival time

```
RMST.difference <-
  function(time, status, group, data, interval = 0.02) {
    #construct surv obj
    event.char <- deparse(substitute(status))
    time.char <- deparse(substitute(time))
    group.char <- deparse(substitute(group))

    time <-data[,time.char]
    event <- data[,event.char]
    arm <- data[,group.char]
    #chk
    if (length(time) != length(event)) {
        stop('ERROR:length(time)!=length(event) ')
      }
    if (is.factor(arm)) {
      arm.level <- levels(arm)
      if (length(arm.level) > 2) {
        stop('more than 2 groups is not limited')
      } else{
        arm <- as.numeric(arm == arm.level[2])
        print(paste0('1-', arm.level[1], ',0-', arm.level[2]))
      }
    }

    #STRT public function  ------------------------------------------------------
--
```

```
calc.maxTau <- function(time, arm) {
  rtn <- min(max(time[arm == 1]),
             max(time[arm == 0]))
  return(rtn)
}
calc.rmst <- function(time, event, arm, tau) {
  diff <-
    rmst2(
      time = time,
      status = event,
      arm = arm,
      tau = tau
    )$unadjusted.result[1,]
  return(c(tau, diff))
}
#recognize change point of significance
change.point <- function(time, sig) {
  rtn <- NULL
  divid <- floor(length(time) / 2)
  for (i in 1:divid) {
    tmp <- sig[(2 * i - 1):(2 * i)]
    if (length(unique(tmp)) == 2) {
      #exist change point and extract the position
      rtn <- c(rtn, 2 * i)
    }
  }
  return(time[rtn])
}
#END public function  -------------------------------------------------------
-
time.list <-
  seq(0.05, calc.maxTau(time = time, arm = arm), interval)

RMST <- NULL
for (tau in time.list) {
  diff <- calc.rmst(
    time = time,
    event = event,
    arm = arm,
    tau = tau
  )
  RMST <- rbind(RMST, diff)
}
#construct data frame for position of change in p value
rmst.chg <- data.frame(RMST)
colnames(rmst.chg) <-
  c('time', 'difference', 'lower', 'upper', 'p')
rmst.chg$category <- ifelse(rmst.chg$p < 0.05,
                            'Significant',
                            'Non-significant')
rmst.chg <<- rmst.chg[!is.na(rmst.chg$category), ]
ss <- change.point(rmst.chg$time, rmst.chg$category)
##plot
ggplot(data = rmst.chg, aes(x = time, y = difference)) +
  geom_line(size = 0.75, alpha = 1,color='#F1A208') +
  geom_ribbon(aes(
    x = time,
    ymax = upper,
```

```
      ymin = lower
    ),
    alpha = 0.5,fill='#F1A208') +
    geom_hline(yintercept = 0,linetype='dashed')+
    xlab(label='Follow-up time')+
    scale_y_continuous('Difference in RSMT',
                       labels=function(x){sprintf("%.2f",x)})+
    theme_bw() +
    theme(
      text = element_text(family = 'serif'),
      axis.title.x = element_text(family = 'serif', vjust = 1.7),
      panel.grid.minor = element_blank(),
      panel.grid.major = element_blank(),
      axis.line = element_line(size = 0.5, colour = "black"),
      legend.background = element_rect(fill = NULL),
      legend.key = element_rect(colour = NA),
      panel.border = element_blank(),
      axis.line.x = element_line(
        size = 0.5,
        linetype = "solid",
        colour = "black"
      ),
      axis.line.y = element_line(
        size = 0.5,
        linetype = "solid",
        colour = "black"
      ))
}
```

Function 5: plotting landmark analysis

```
landmark <- function(time, status, group, data,
                     timelist,legend.poc=c(0.2,0.2)) {

  #chk parameter
  if (length(timelist) < 1) {
    stop('A time point must be specficed and can not be ZERO')
  }
  #construct surv obj
  event.char <- deparse(substitute(status))
  time.char <- deparse(substitute(time))
  group.char <- deparse(substitute(group))
  fml <-
    as.formula(paste0('Surv(', time.char, ",", event.char, ")~", group.char))
  data <- data

  single <- function(landpoint) {
    #remove rows who have missing value in key variable
    data.tmp1 <-
      data[complete.cases(data[, c(event.char, time.char, group.char)]), ]
    data.tmp1[data.tmp1[, time.char] > landpoint, event.char] <- 0
    #Post-landmark
    data.tmp2 <-
      data[complete.cases(data[, c(event.char, time.char, group.char)]), ]
```

```
  data.tmp2 <- data.tmp2[data.tmp2[, time.char] > landpoint, ]
  #add p value from landmark analysis
  preland <- survdiff(as.formula(fml), data.tmp1)
  preland.pvalue <- pchisq(preland$chisq, length(preland$n) - 1,
                           lower.tail = FALSE)
  #Post-landmark
  postland <- survdiff(as.formula(fml), data.tmp2)
  postland.pvalue <-
    pchisq(postland$chisq, length(postland$n) - 1,
           lower.tail = FALSE)
  return(c(landpoint, preland.pvalue, postland.pvalue))
}

rtn <- data.frame(t(sapply(timelist, single)))
colnames(rtn) <- c('time', 'pre_landmark', 'post_landmark')
rtn$pre_landmark <- -log10(rtn$pre_landmark)
rtn$post_landmark <- -log10(rtn$post_landmark)
new <-
  rbind(
    data.frame(
      time = rtn$time,
      logp = rtn$pre_landmark,
      group = 'Pre-landmark analysis'
    ),
    data.frame(
      time = rtn$time,
      logp = rtn$post_landmark,
      group = 'Post-landmark analysis'
    )
  )

p <- ggplot(data = rtn) +
  geom_errorbar(
    aes(x = time, ymin = pre_landmark, ymax = post_landmark),
    alpha = 1,
    width = 0.1,
    size = 0.5,
    color = '#D4F4DD'
  ) +
  geom_point(data = new,
             aes(x = time, y = logp, color = group),
             alpha = 1) +
  geom_hline(yintercept = -log10(0.05), linetype = 'dashed') +
  scale_color_manual(values = c('#0E7C7B', '#D62246')) +
  xlab(label = 'Follow-up time') +
  scale_y_continuous(
    '-log10(p value)',
    labels = function(x) {
      sprintf("%.2f", x)
    }
  ) +
  theme_bw() +
  theme(
    text = element_text(family = 'serif'),
    axis.title.x = element_text(family = 'serif', vjust = 1.7),
    panel.grid.minor = element_blank(),
    panel.grid.major = element_blank(),
    axis.line = element_line(size = 0.5, colour = "black"),
```

```
      legend.background = element_blank(),
      legend.key = element_blank(),
      legend.title = element_blank(),
      legend.direction = "horizontal",
      legend.position = legend.poc,
      panel.border = element_blank(),
      axis.line.x = element_line(
        size = 0.5,
        linetype = "solid",
        colour = "black"
      ),
      axis.line.y = element_line(
        size = 0.5,
        linetype = "solid",
        colour = "black"
      )
    )
  return(p)
}
```

Function 6: plotting time-varying HR

```
timevarying.HR <- function(time, status, group,
           data,df = 4,nsmo = 40) {
    #construct cox regression
    event <- deparse(substitute(status))
    time <- deparse(substitute(time))
    group <- deparse(substitute(group))
    fml <- paste0('Surv(', time, ",", event, ")~", group)
    sfit <- coxph(as.formula(fml), data)
    averageHR <- exp(sfit$coefficients)

    x <- cox.zph(sfit, transform = 'identity')
    xx <- x$x #raw time without transformation
    yy <- x$y #residual

    pred.x <- seq(from = min(xx),
                  to = max(xx),
                  length = nsmo)
    temp <- c(pred.x, xx)
    lmat <- ns(temp, df = df, intercept = TRUE)
    pmat <- lmat[1:nsmo,]       # for prediction
    xmat <- lmat[-(1:nsmo),]

    #clear missing element
    #coefficient and 95% CI --- not the HR value
    keep <- !is.na(yy)
    qmat <- qr(xmat[keep,])
    yhat <- pmat %*% qr.coef(qmat, yy)
    bk <- backsolve(qmat$qr[1:df, 1:df], diag(df))
    xtx <- bk %*% t(bk)
    seval <- ((pmat %*% xtx) * pmat) %*% rep(1, df)
    SE <-  sqrt(x$var[1, 1] * seval)
    yup <- yhat + 1.96 * SE
    ylow <- yhat - 1.96 * SE
```

```r
  #create data frame for plotting
  #exp transformation to get HR and 95% CI
  hr.chg <-
    data.frame(
      time = pred.x,
      point = exp(yhat),
      upper = exp(yup),
      lower = exp(ylow)
    )
  colnames(hr.chg) <- c('time', 'point', 'upper', 'lower')
  p <-  ggplot(data = hr.chg, aes(x = time, y = point)) +
    geom_line(size = 0.75,
              alpha = 1,
              color = '#58355e') +
    geom_ribbon(aes(x = time,
                    ymax = upper,
                    ymin = lower),
                alpha = 0.2,
                fill = '#58355e') +
    geom_hline(yintercept = 1, linetype = 'dashed') +
    xlab(label = 'Follow-up time') +
    scale_y_continuous(
      'Hazard ratio',
      trans = 'log',
      breaks = trans_breaks("log2", function(x)
        2 ^ x),
      labels = function(x) {
        sprintf("%.2f", x)
      }
    ) +
    theme_bw() +
    theme(
      text = element_text(family = 'serif'),
      axis.title.x = element_text(family = 'serif', vjust = 1.7),
      panel.grid.minor = element_blank(),
      panel.grid.major = element_blank(),
      axis.line = element_line(size = 0.5, colour = "black"),
      legend.background = element_rect(fill = NULL),
      legend.key = element_rect(colour = NA),
      panel.border = element_blank(),
      axis.line.x = element_line(
        size = 0.5,
        linetype = "solid",
        colour = "black"
      ),
      axis.line.y = element_line(
        size = 0.5,
        linetype = "solid",
        colour = "black"
      )
    )
  return(p)
}
```

An example: Run 6 functions and combine the 6 plots

```
library(rms)
library(survRM2)
library(ggplot2)
library(plyr); library(dplyr)
library(cowplot)

options(digits = 3)
options(scipen = 999)
# data construction ----------------------------------------------------------
IPD <- KM_data_2group(df1='./image_4/1-km.csv',
                      df2='./image_4/2-km.csv',
                      nr1='./image_4/1-num.csv',
                      nr2='./image_4/2-num.csv',
                      tot.event1=188, tot.event2=126,
                      arm1='intervention', arm2='control')
#define Group 1-group of interest 0-control
IPD$group <- (IPD$group=='intervention')*1

max(IPD$time)
xmax <- max(IPD$time)

# KM plot -------------------------------------------------------------------
P1 <- KM_plot(status=status,time=time,group=group,data=IPD,
              HR.loc=c(20,0.7),PH=TRUE,PH.loc=c(20,0.9))+
  scale_x_continuous(limits=c(0,xmax))

# difference in two Kaplan-Meier estimates ---------------------
P2 <- SurvDiff.surv(
  time = time,  status = status,  group = group,
  data = IPD,  converse=FALSE
)+
  scale_x_continuous(limits=c(0,xmax))
# risk difference ----------------------------------------------
P3 <- RiskDiff.surv(
  time = time,  status = status,  group = group,  data = IPD,
  method = 'Pse',
  timelist = timelist,  boot.num = 100
)+scale_x_continuous(limits = c(0,xmax))
# difference in RMST ------------------------------------
P4 <- RMST.difference(
  time = time,  status = status,  group = group,  data = IPD,
  interval = interval
) + scale_x_continuous(limits = c(0,xmax))

# landmark analysis -------------------------------------------------
P5 <- landmark(
  time = time,  status = status,  group = group,  data = IPD,
  timelist=seq(3,34,1),legend.poc=c(0.3,0.1)
  )+ scale_x_continuous(limits = c(0,xmax))

# time-varying hazard ratio -------------------------------------------------
```

```
P6 <-
  timevarying.HR(
    time = time,status = status,group = group,data = IPD,
    df = 3, nsmo = 100
  )+ scale_x_continuous(limits = c(0,xmax))


# -------------------------------------------------------------------------------
-#
ALL <- plot_grid(
  P1,P4,P2,P5,P3,P6,
  align = 'v',  nrow = 3,  ncol = 2,
  labels =LETTERS[1:6]
)
ggsave(
  'figure.tif',  ALL,
  device = 'tiff',  width = 15,  height = 10,  dpi = 600,  compression = "lzw"
)
```
